# Nanoenabled Trainable Systems: From Biointerfaces to Biomimetics

**DOI:** 10.1021/acsnano.2c08042

**Published:** 2022-12-14

**Authors:** Pengju Li, Saehyun Kim, Bozhi Tian

**Affiliations:** † Pritzker School of Molecular Engineering, The University of Chicago, Chicago, Illinois 60637, United States; ‡ Department of Chemistry, The University of Chicago, Chicago, Illinois 60637, United States; § The James Franck Institute, The University of Chicago, Chicago, Illinois 60637, United States; ∥ The Institute for Biophysical Dynamics, University of Chicago, Chicago, Illinois 60637, United States

**Keywords:** Trainable biointerfaces, nanomaterials, biomimetics, living materials, adaptive systems

## Abstract

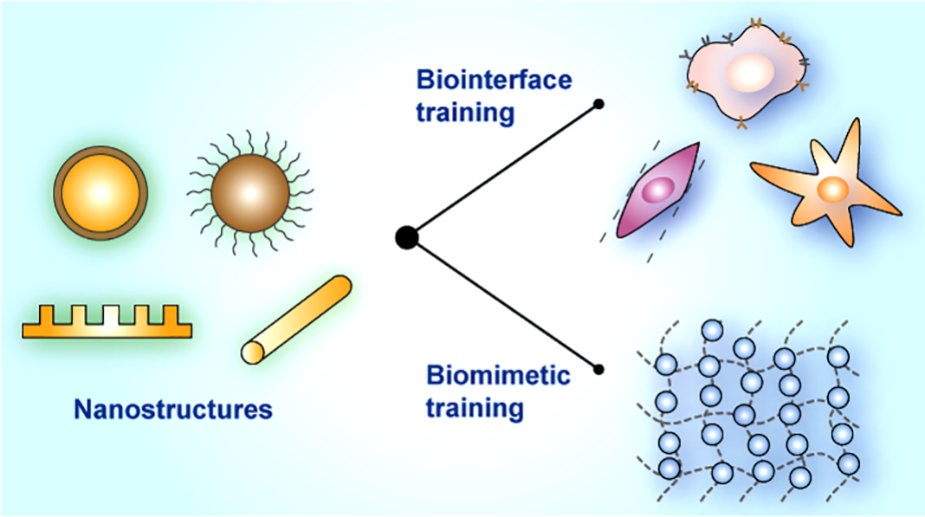

In the dynamic biological system, cells and tissues adapt to diverse environmental conditions and form memories, an essential aspect of training for survival and evolution. An understanding of the biological training principles will inform the design of biomimetic materials whose properties evolve with the environment and offer routes to programmable soft materials, neuromorphic computing, living materials, and biohybrid robotics. In this perspective, we examine the mechanisms by which cells are trained by environmental cues. We outline the artificial platforms that enable biological training and examine the relationship between biological training and biomimetic materials design. We place emphasis on nanoscale material platforms which, given their applicability to chemical, mechanical and electrical stimulation, are critical to bridging natural and synthetic systems.

Adaptability in biological systems allows cells and tissues to acquire improved or different capabilities through exposure to, or “training” by, external stimuli.^1^ In this instance, training refers to the process by which a change is produced in a system’s properties that persists even after the stimulus is removed, altering the system’s future behavior. In biological systems, training examples include the hardening of muscle and bone under cyclic loading or the stimuli-induced differentiation of stem cells that leads to permanent changes in their properties.^2^ The process of training requires structural and molecular reconfiguration within and outside of cells, as well as genetic and epigenetic modifications to create memory.^3^ Conventional approaches to material system designs, where design parameters are fixed once identified, have limited scope in creating intelligent materials systems that yield such dynamic reconfiguration and adaptable material properties. As such, a deeper understanding of the mechanisms governing cell response to stimulation and adaptive behavior is crucial to the design of biomimetic materials.^4,5^

Cells in an organism are exposed to many environmental cues, including chemical and biomolecular species, mechanical stress, and bioelectrical signals. The molecular machinery of biological cells allows them to sense these cues and dynamically modify their properties according to the environment. In the adaptive immune system, for example, antigen exposure leads to the activation of chemical signaling pathways that determine T-cell and B-cell migration, differentiation, and proliferation. Additionally, epigenetic reprogramming prepares the cell to react faster and stronger to reinfection, while genetic recombination of receptor sequences encodes long-term antigen-specific memory in memory cells.^6^ Cells of the innate immune system, such as natural killer cells and macrophages, are also capable of carrying immunologic memory, which provides nonspecific immunity against a range of pathogens.^3,7,8^ Detection of pathogen- or damage-associated molecular patterns (PAMPs and DAMPs) stimulates epigenetic and metabolic reprogramming within the cells, altering gene transcription for innate immune responses. These changes, which persist from months to a few years, impart a memory to innate immune cells that increases their sensitivity to nonspecific immune challenges.

Mechanosensitive ion channels and focal adhesion sites control cell shape, alignment, and differentiation and tissue dynamics.^9−12^ Upon sensing mechanical cues, focal adhesion sites assemble and mechanotransduction occurs through matrix–integrin–cytoskeletal interactions, which regulate focal adhesion kinase (FAK), SRC-family kinases, and RHO-GTPases.^13^ Following cytoskeleton remodeling, activated YAP/TAZ transcription factors interact directly with DNA to influence gene expression.^13−15^ Mechanical stress causes mechanosensitive ion channels to open, and the resulting calcium influx modulates contraction *via* calmodulin/caldesmon interactions.^9^ Structural reorganization of extracellular matrix (ECM) fibers, which triggers the hardening of fibrous biological components such as collagen, fibrin, and actin networks under cyclic deformation, can also lead to mechanical adaptation.^16−19^ It has also been shown that muscles can be strengthened by repeated mechanical training, which increases cell nuclei numbers, muscle mass, metabolic activity, and the preferential alignment of cells with their ECM.^20,21^

Bioelectric signals, such as action potentials, regulate cardiac rhythm and neuron activity. During rest, cells are polarized with a membrane potential of −70 mV. When an electrical stimulus is applied to the membrane, an action potential is fired through the cellular circuits.^22^ Electrically gated ion channels allow Na^+^ to flow in and K^+^ to flow out to maintain membrane polarity. Bioelectric stimulation has been studied extensively in electrically excitable cells, including neurons and cardiomyocytes.^23,24^ In neurons, trainable behavior is modulated by glutamate-gated ion channels (AMPA) and calcium influx. Calcium elevation in the cytosol promotes synaptic enhancement and activation of the transcription factor CREB, which regulates long-term potentiation and memory consolidation.^25^ Additional dendritic spines and synaptic connections, as well as neurons, contribute to creation of a long-term memory. Rhythmic beating of the heart is mediated by pacemaker cells located in the sinoatrial and atrioventricular nodes.^26^ Excitation–contraction coupling occurs when action potentials propagate through gap junctions to cause mechanical contractions.^27^ This process involves Ca^2+^ influx during depolarization and calcium-induced calcium release from intracellular organelles such as mitochondria.^28,29^ Upon activation of ATP on the myosin head, Ca^2+^ binding to cardiac troponin-C frees myosin-bonded actin, and allows it to move toward the sarcomere center.^30^ Relaxation occurs as Ca^2+^ is removed by the sarcoplasmic reticulum. Electrical training of cardiomyocytes, however, is rare and less well understood. Our group recently developed nanostructured capacitor-like electrodes for training cardiomyocytes at subthreshold voltages.^31^ While the exact mechanisms by which cardiomyocytes respond to electrical training are unknown, Ca^2+^ level regulation, cytoskeleton structural adaptation, and the dynamics of mitochondria (which account for a third of the volume of mature cardiomyocytes) deserve special consideration.

Conventional material design is a one-way journey wherein material functions are fixed by the initial design parameters. Consequently, the system maintains the same properties throughout its lifetime rather than dynamically adapting to real-world environmental cues such as temperature, humidity, light, and pressure, which change according to place and time. An inability to adapt can result in energy-inefficient applications and unexpected material failures.^32^ For example, traditional polymers such as polyethylene bags are easily damaged by mechanical stress. The US alone produces 104 million metric tons of CO_2_ equivalent annually from such major commodity polymers. A self-adapting polymer that can detect and repair unexpected damage would extend the polymer’s life cycle and reduce the energy cost of its production.^33^ Therefore, synthetic materials that mimic biologically trainable models will allow the system to better cope with environmental changes through reprogramming, autoregulation, and self-correction.^34^

Nanomaterials play an important role in the design of biomimetic training systems. The benefits offered by nanomaterials in biointerface modulation, which have been discussed in previous reviews, can be extended to artificial trainable systems.^35^ First, scaling the building blocks to the nanometer scale, at the order of which electrical, mechanical, and chemical energy amplitudes converge, results in efficient energy transduction.^36^ Nanomaterials can serve as transducers that convert macroscale signals into cues that are interpreted by cellular and material systems to guide training. Second, nanomaterials are size-matched to biological components, which enables them to mimic certain biological functions.^37^ For example, silicon nanowires that can be internalized by cells can regulate cell migration, essentially mimicking the activity of the cytoskeleton.^38,39^ Functional nanomaterials that emulate biological components can also be integrated into a synthetic matrix and used to create biomimetic systems. A further advantage of nanomaterials is that they are capable of dynamic reconfiguration in response to external stimuli and can be fine-tuned to exhibit spatiotemporal heterogeneity, a property crucial for adaptive evolution when faced with sudden changes. Finally, because nanostructures have a high surface-to-volume ratio, they can be functionalized through chemical grafting, adding yet another dimension to training.

Here, we introduce trainable biointerfaces and biomimetic systems based on nanostructured platforms (Figure 1). We discuss chemical, mechanical, and electrical approaches to biointerface training. We review the important role of synthetic nanomaterials in biological training and how biological training principles are applied for design of biomimetic smart training systems. Additionally, we identify the advantages and limitations of current efforts and forecast future directions, in which we anticipate a synergy between advanced nanomaterial synthesis, artificial intelligence, synthetic biology, and engineered living materials (ELMs). A summary of representative nanomaterials and their roles in biological and biomimetic trainings is also provided in the Table 1.

**Figure 1:**
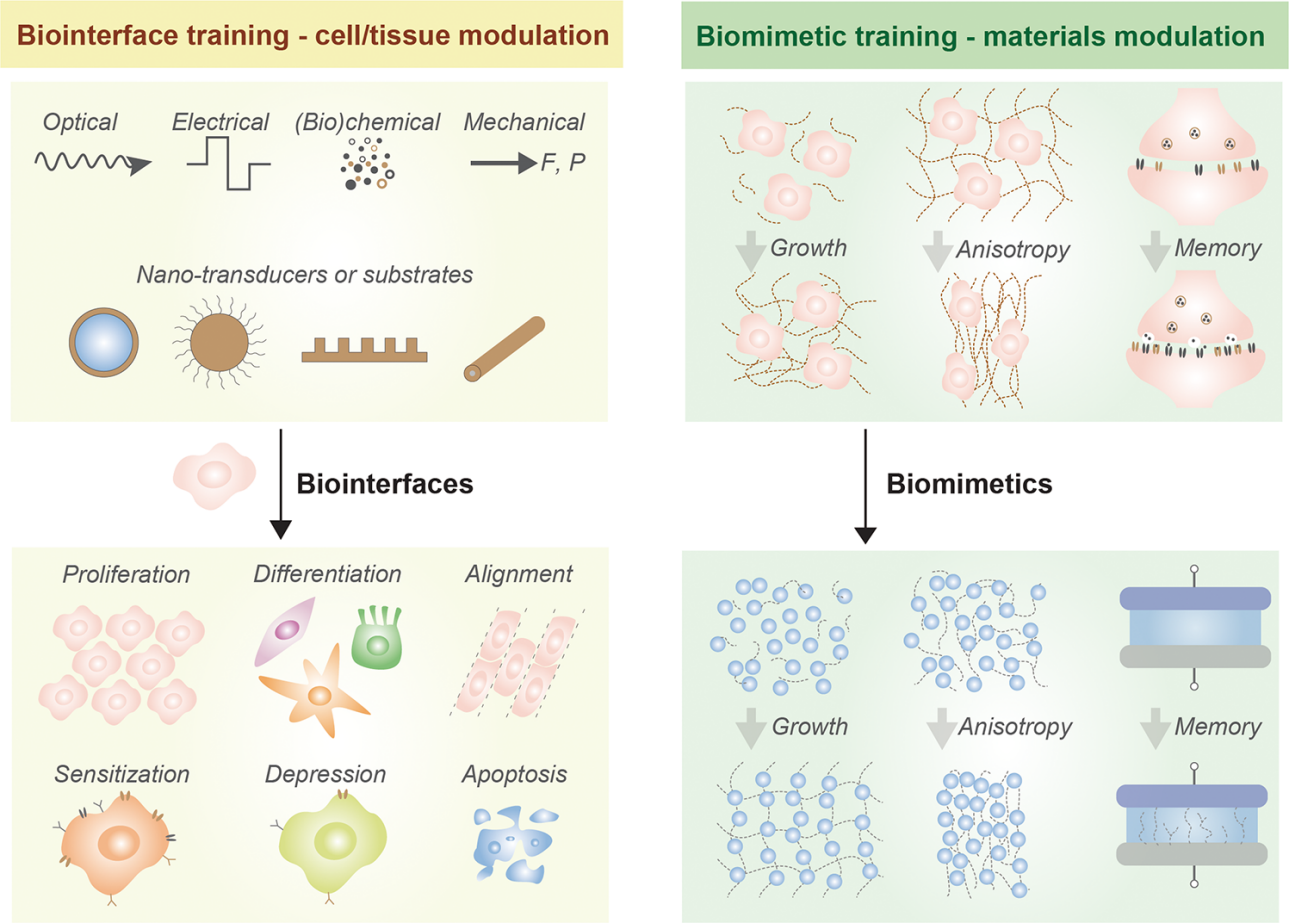
Nanoenabled trainable systems. (left) Nanomaterials act as signal transducers for cell training, leading to various cell behaviors and fates. (right) Biomimetic trainable systems enabled by nanomaterials yield triggered-growth, anisotropic adaptation, and memory formation.

**Table 1:** Representative Nanomaterials Used for Biointerface and Biomimetic Training

type	nanomaterial	role of nanomaterial	mechanism	results of training	speculated duration	reversibility
**Biointerface Training**						
biochemical	nanocarriers composed of lipid, polymer, protein, and virus^42−46^	drug-delivery vehicle enclosing or coated with bioactive molecules	genetic, epigenetic, and metabolic reprogramming of immune cells	faster and more vigorous immune response toward subsequent infection	months to life-long	reversible & irreversible
biochemical	stimuli responsive nanoparticle carriers^49−51^	enable controlled and site-specific presentation of bioactive cargo				
mechanical	nanotopological substrate^52−60^	substrate for cell growth that provides persistent mechanical cues	mechanosensing & transduction through focal adhesion sites or mechanosensitive ion channels	stem cell differentiation	life-long	irreversible
mechanical	stimuli-responsive nanoparticles, nanotubes, and nanowires^37,61,64^	transduce physical stimuli (*i.e.*, light, magnetic field) into a mechanical force				
electrical	nanoporous microelectrode^31^	improve charge injection within safe voltage range and form good electrical coupling with cells	possibly due to alteration of cell resting membrane potential during electrical training	synchronized cardiomyocyte contraction with suprathreshold stimuli	transient	reversible
electrical	semiconductor nanoparticles^68^	deliver photogenerated currents that enabled cardiac tissue training		synchronized heart contraction with suprathreshold stimuli		
**Biomimetic Training**						
mechanical	nanofibers and nanocrystalline domains^71−75^	mimic fibrous biological components such as collagen, fibrin, and actin networks	structural reorganization and alignment along the direction of strain	hardening change in resistance	life-long	reversible & irreversible
mechanical	piezoelectric nanoparticles^77,78^	mimic biological cells that sense and transduce force and synthesize matrix using simple monomers	generate electrons that lead to polymerization	hardening	life-long	irreversible
electrical	conductive nanofilament^86^	mimic synaptic conditioning which enables on/off logic gates	applied voltage bias induces electrochemical reduction of nanofilaments	resistance switch and formation of logic gates	life-long	reversible
electrochemical	semiconducting polymers^88−94^	mimic synaptic conditioning by enabling efficient doping/undoping of ions that modulate channel capacitance	applied voltage bias induces ionic injection into semiconducting polymers	artificial spiking and synaptic conditioning	life-long	reversible

## Current Progress

### Nanomaterial Systems for Biointerface Training

Nanomaterial-based biointerface training offers improved signal transduction, versatility, controllability, and specificity from the single-cell level to tissue- and organ-level, compared to conventional bulk platforms. In biochemical training, for example, nanocarriers can be selectively released inside target cells, according to cell phenotype. In mechanical or electrical training, nanostructured topology forms seamless interfaces with cells or tissues, leading to enhanced signal transduction. Functionalized nanoparticles are particularly suited for single-cell or subcellular level training. In the following sections, we discuss the recent development of nanoenabled biochemical, mechanical, and electrical training systems that interact with a variety of biological targets.

#### Nanomaterial-Enabled Biochemical Training

Innate immunity can be modulated by inhibitors (*i.e.*, β-hydroxybutyrate, vorinostat), promotors (*i.e.*, mevalonic acid, uric acid), nucleic acid drugs (mRNA, siRNA, and lncRNA), immunostimulatory polymers (chitosan, peptidoglycan, and hyaluronan), immunoregulatory proteins (antibodies such as IL-1β and GM-CSF), and PAMPs.^40,41^ The nanomedicine delivery platform is determined by the physicochemical properties of the bioactive cargo. Liposomes efficiently encase hydrophilic payloads in their aqueous core, while micelles and emulsions based on amphiphilic lipids are better suited for hydrophobic payloads.^42,43^ Viral vectors are ideally suited to carrying nucleic acid drugs.^44^ Once a suitable delivery structure is identified, the size and surface modifications of the bioactive nanomedicine cargo can be further tuned for efficient and targeted delivery to innate immune cells.^45,46^

**Figure 2:**
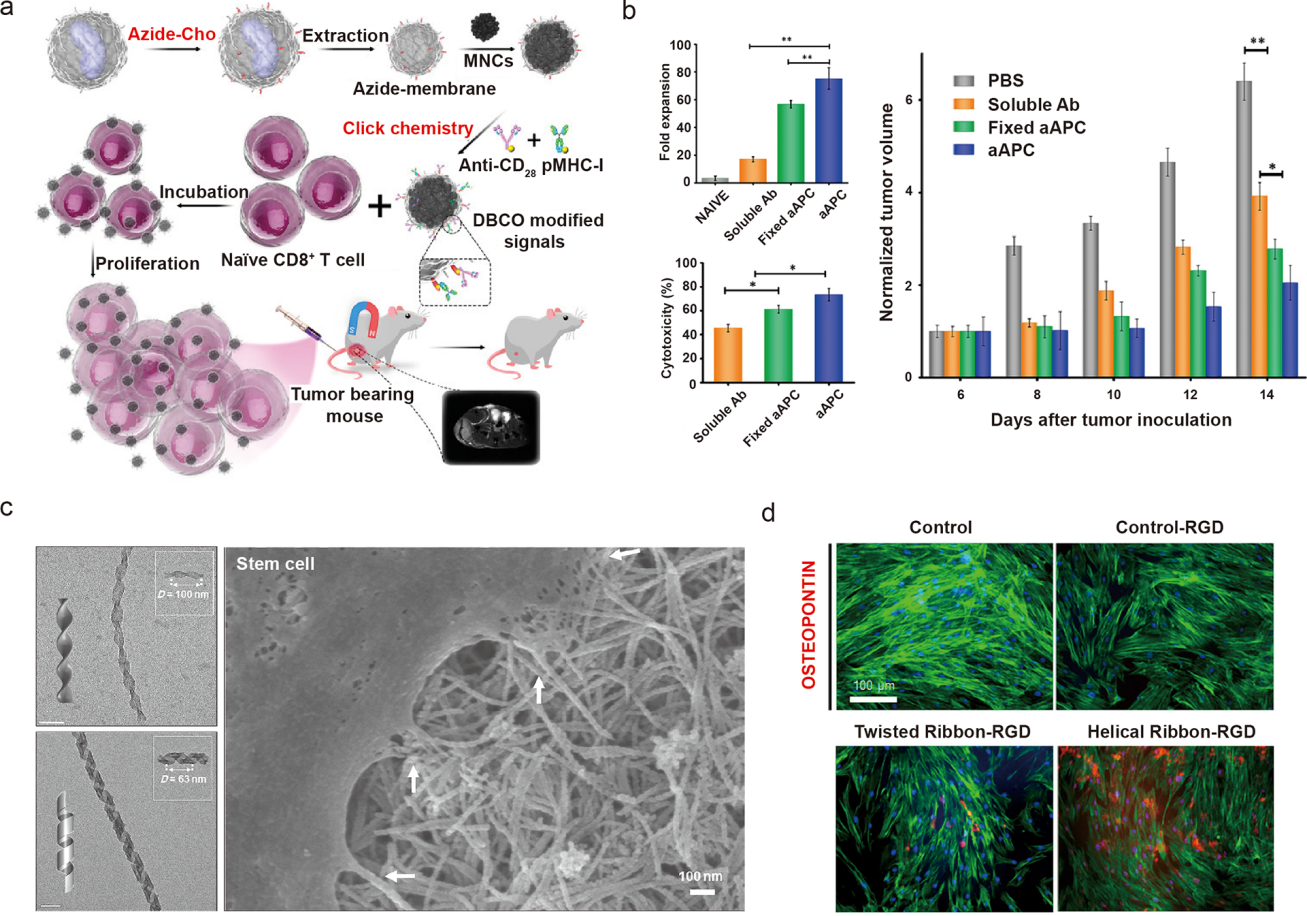
Cell training *via* nanomaterial-mediated biochemical (a, b) and mechanical (c, d) stimuli. (a) Schematic depicting the use of pMHC-I and anti-CD28 decorated magnetic nanoclusters (MNCs) as artificial antigen presenting cells (aAPCs) to stimulate T cells *in vitro* and promote antitumor activity in mice. aAPC-decorated T cells can be localized to EG-7 tumor cells *in vivo* using a magnetic field. (b) Compared to soluble antibody (Ab), T cells stimulated by aAPCs show improved proliferation and cytotoxicity against tumor cells, demonstrating the effectiveness of nanomediated biochemical cues in cell training. Panels a and b are adapted with permission from ref (47). Copyright 2017 American Chemical Society. (c) Transmission electron microscopy (TEM) image of (top left) helical silica nanoribbon and (bottom left) twisted silica nanoribbon, and (right) scanning electron microscope (SEM) image of stem cells cultured on RGD ligand-decorated helical nanoribbon. (d) Immunofluorescence staining shows enhanced osteopontin expression (red) in cells cultured on helical ribbon-RGD, suggesting osteogenic differentiation. Panels c and d are adapted with permission from ref (54). Copyright 2013 American Chemical Society.

Adaptive immune cells can be trained through biochemical stimuli. Adoptive T-cell therapy, using nanoparticles decorated with antigens or carrying bioactive payloads (Figure 2a,b), can train T cells to become tumor-specific or downregulate immunosuppressive pathways, increasing cancer specificity and cytotoxicity when administered to patients.^47^ Antigen-carrying nanovaccines can be used to train B cells so that memory B cells proliferate and differentiate into plasma cells when they re-encounter the antigen.^48^

Stimuli-responsive nanoparticles allow spatiotemporal control over the delivery of biochemical cues. Bioresponsive nanoparticles that degrade in response to lysosomal pH, enzymes, or reductive cytosol ensure effective antigen release upon uptake by dendritic cells. The antigen is subsequently presented to naive T cells to elicit training.^49^ Biodistribution can be controlled using external magnetic fields. Li *et al*. decorated Fe_3_O_4_ magnetic nanoclusters with tumor antigens and used a magnetic field to target the decorated nanoclusters to lymph nodes, the reservoir of immune cells. Antigens were taken up effectively by dendritic cells, and T cells proliferated with enhanced cytotoxic activity and clonal diversity.^50^ Additionally, by harnessing the ability of nanoparticles to absorb or scatter light, nanoparticle-mediated photothermal-, photodynamic-, and radio-therapy can kill cancer cells *in vivo* and release *in situ* anticancer vaccines (DAMPs and tumor antigens) that induce training of the innate and adaptive immune system.^51^

#### Nanomaterial-Enabled Mechanical Training

Cells can sense nanoscale mechanical variations in their extracellular matrix (ECM). Synthetically patterned nanotopological substrates can deliver targeted mechanical stimulation, via mechanotransduction,^9,11,12^ to regulate cell shape, alignment, adhesion, and differentiation. Nanotopological substrate features, including diameter, spacing, height, geometry, and periodicity, collectively determine stem cell fate.^52^ Nanopillar arrays of silicon, with pillars of 100 nm diameter (versus 50 nm), promoted osteogenic differentiation of human mesenchymal stem cells (hMSCs). Smaller spacing between nanopillars resulted in better differentiation of hMSCs from young donors compared to hMSCs from older donors.^53^ hMSCs differentiated into osteoblasts when cultured on helical silica nanoribbons of 63 nm periodicity, but twisted silica nanoribbons of 100 nm periodicity did not lead to osteoblast commitment (Figure 2c,d).^54^ Osteogenic differentiation of the macrophage cell line RAW264.7 occurred preferentially on nanopillar patterns compared with grooves and holes.^55^ Nanotopological cues may also encourage cells to dedifferentiate and transdifferentiate.^56,57^ The mechanical properties of nanostructured substrates play a substantial role in guiding stem cell fate. MSC differentiation into neurons, osteoblasts, or adipocytes is enhanced by nanostructured substrates with stiffnesses that mimic their natural ECM. Osteogenic differentiation is promoted at a high Young’s modulus of 25–40 kPa, while differentiation into neurons is promoted at 0.1–1 kPa.^58^ The cells are sensitive to nanoscale variations in topography, as integrin-binding ECM ligands spaced below a threshold of 50–70 nm enable clustering and activation of integrins.^59,60^ It is important to note, however, that cell fate cannot be predicted based purely on the nanotopological design of the material.

Other physical cues, such as magnetic fields and temperatures, can be translated into mechanical signals by responsive materials. For example, one study conjugated superparamagnetic iron oxide nanoparticles with RGD ligands and tuned the oscillation of the ligands using a magnetic field. Low oscillation frequencies promoted nuclear localization of YAP/TAZ in stem cells, which influenced cell differentiation.^61^ In another study, fibroblasts were grown on a thermoresponsive shape memory film with temporary parallel nanogrooves designed perpendicular to permanent nanogrooves. Once fibroblasts aligned parallel to the temporary nanogrooves, heat-treatment-induced shape recovery shifted the film pattern 90°, returning the pattern to the original grooves. Although cell alignment did not change immediately, the cells gradually remodeled their cytoskeleton to realign themselves along the altered pattern of direction.^21^ These studies show that responsive materials may be utilized to control the timing of mechanical cues and the behavior of cells.

The photoacoustic effect can convert light into mechanical stimuli. Depending on the laser parameters, photoacoustic stimulation may generate tunable pressure waves with wavelengths at the cellular and subcellular levels.^62^ As a result, mechanosensitive machinery on the cell membrane could be perturbed, leading to mechanotransduction and changes in the transcription profile of bone-specific genes.^63^ Green *et al*. demonstrated that osteogenesis was significantly promoted when multipotent marrow stromal cells were treated daily for 16 days with nanoparticle-enhanced photoacoustic stimulation for 10 min.^64^ Another group demonstrated that photoacoustic stimulation mediated by graphene oxide/poly(lactic-*co*-glycolic acid) composite increased alkaline phosphatase activity, calcium concentration, and osteopontin expression in bone marrow mesenchymal stem cells.^65^ Using the photoacoustic effect, silicon nanowires taken up by human umbilical vein endothelial cells can break apart surrounding microtubules, altering cytoskeletal structures.^37^

With ultrasonication, one can effectively control the timing of mechanical stimulation. In neurons expressing the mechanosensitive MscL channel, ultrasonication activated opening of the MscL nanovalve and allowed precise firing of action potentials within milliseconds.^66^ Modulation of neuron activity *via* ultrasonication can also influence organism-level behavior; ultrasonic stimulation in *Caenorhabditis elegans* neurons expressing TRP-4, a pore-forming subunit of a mechanotransduction channel, resulted in a change in locomotory behavior.^67^

#### Nanostructured Electrode-Enabled Bioelectrical/Electrochemical Cell Training

**Figure 3:**
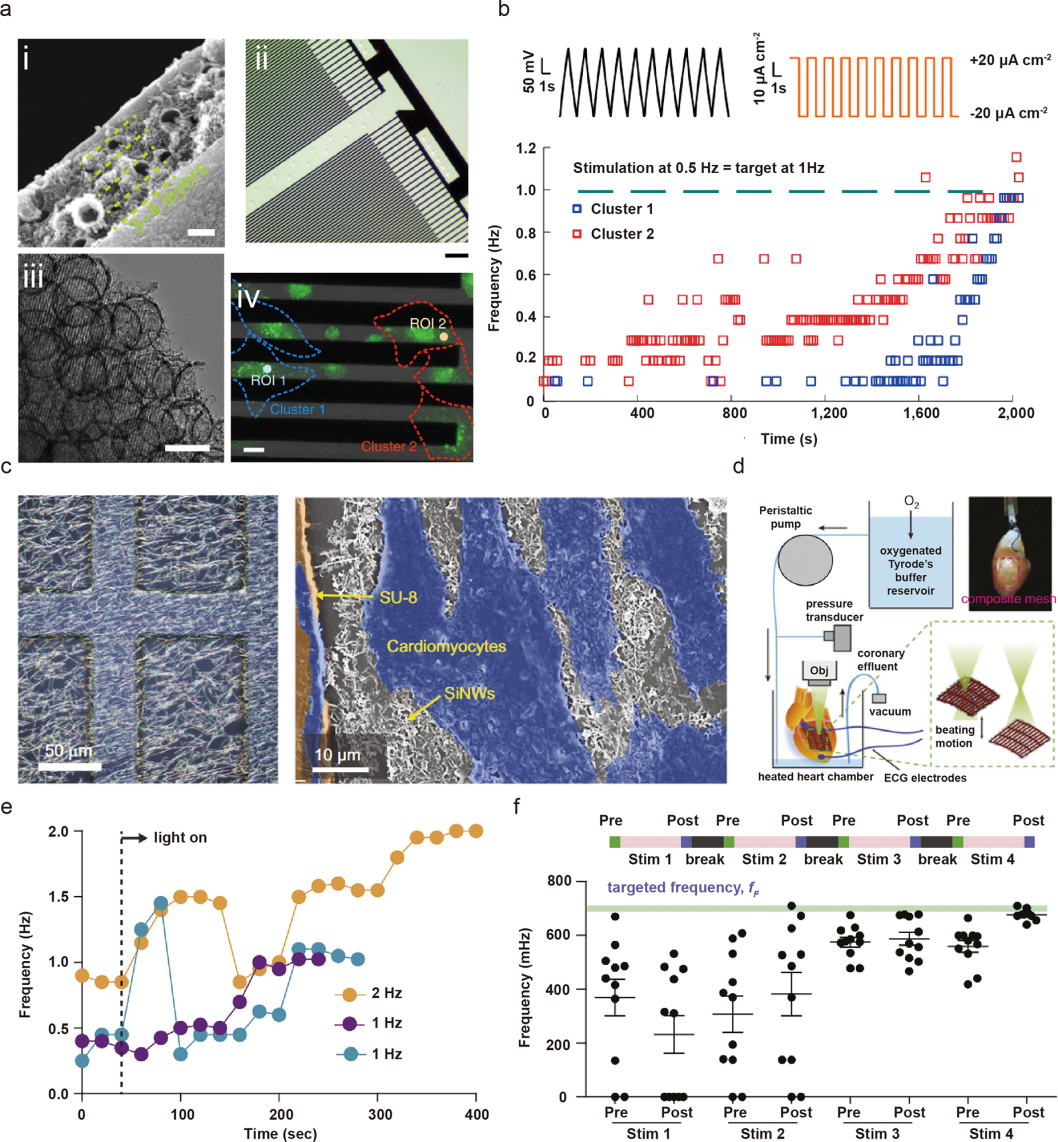
Nanostructured electrode and photoelectrode for cardiac training. (a) (i) Cross-sectional SEM image of nanoporous carbon electrode. (ii) Optical microscopy of the interdigitated electrode design. (iii) TEM image showing the porous structure. (iv) Fluorescence imaging showing cardiomyocytes grown on the electrode. (b) Electrical training enables synchronous pacing of cardiomyocytes at different regions of interest. Panels a and b are adapted with permission from ref (31). Copyright 2021 Springer Nature. (c) Optical images showing silicon nanowires on SU-8 mesh and interfaces with cardiomyocytes. (d) Set up of *ex vivo* isolated heart stimulation. (e, f) Optoelectronic stimulation enables (e) isolated heart training and (f) cardiomyocyte training toward desired frequencies. Panels c–f are adapted with permission from ref (68). Copyright 2019 National Academy of Science.

Electrodes with nanoscale topologies provide seamless electrophysiological coupling for stimulation. In comparison to conventional parallel-plate or wired electrodes, micropatterned supercapacitor-like electrodes produce a more effective electrical field that is more uniform across the entire device area. Consequently, cells at different regions of interest experience a similar coherent electrical field for pacing, a prerequisite for training spatially distributed cells. The enhanced electrochemical properties offered by nanostructured topology also result in increased pure-capacitive currents within safe voltage limits for the prevention of water electrolysis and ROS generation that could be harmful to cells. Fang *et al*. investigated the adaptation of cardiomyocytes to subthreshold currents using interdigitated nanoporous carbon electrodes (Figure 3a).^31^ Cardiomyocytes did not initially respond to subthreshold amplitudes. However, the cells adapted to the pacing frequency with synchronous contractions when electrical pulses were persistently supplied (∼1900 s, 1 Hz) (Figure 3b). These results suggest the process “retrained” the cardiomyocytes to become more sensitive to subthreshold stimuli, possibility *via* changes in resting membrane potentials and stochastic activities within the cells. Using photoelectric modulation, the same training principle has also been demonstrated. Parameswaran *et al*. showed that coaxial p-type/intrinsic/n-type (PIN) silicon nanowires enable neuromodulation and cardiomyocyte modulation.^68,69^ Using silicon nanowire networks on freestanding polymer grid substrates, the authors achieved high-density arrays of nanostructured photoelectrodes, which could be modulated with high spatial resolution and targeted to specific cells (Figure 3c). In optical training of *in vitro* cardiomyocytes, and an ex vivo isolated heart, the beating frequency gradually adapted to the light pulse frequency (Figure 3d–f). Cardiomyocyte training also revealed a possible “memory effect” when the break time between stimulations was less than 10 min.

### Nanomaterial Systems As Adaptive or Trainable Systems

#### Mechanically Trainable Nanomaterials

**Figure 4:**
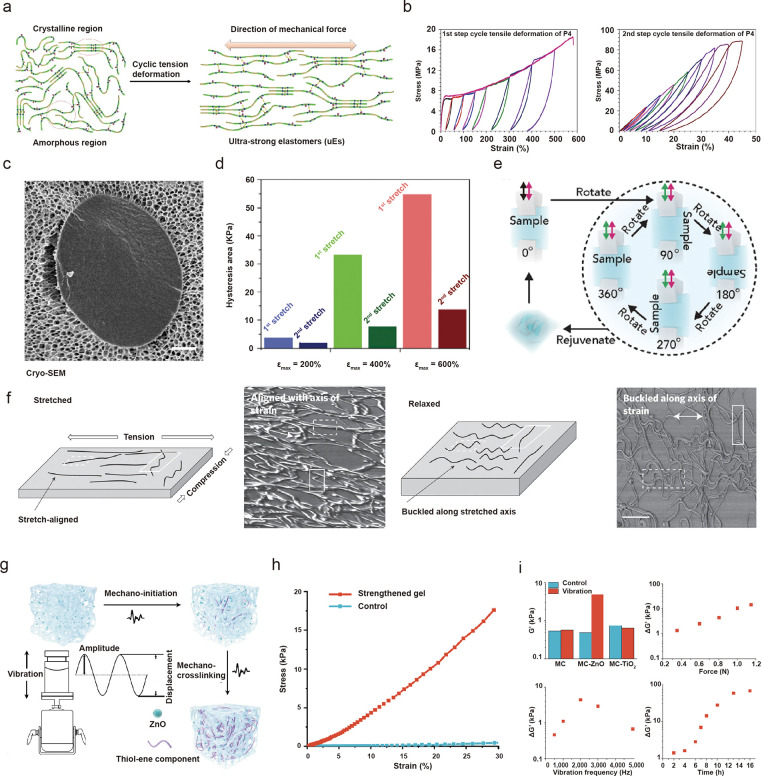
Mechanically trainable and adaptable nanomaterial–hydrogel composites. (a) Cyclic deformation of polyamide elastomer results in alignment of nanocrystalline domains along the tensile direction and (b) stress–strain hysteresis resembling the Mullins effect. Panels a and b are adapted with permission under a Creative Commons CC BY License from ref (72). Published 2019 by Springer Nature. (c) Starch granule embedded in nanoporous polyacrylamide–alginate (PAA-Alg) hydrogel matrix. (d) Starch/PAA-Alg granular composite shows strain-history-dependent energy dissipation. (e) Strain-history encoded in the granular composite can be erased by rotation and stretching at different angles. Panels c and d are adapted with permission from ref (74). Copyright 2020 Elsevier. (f) Stretching induces the alignment of CNTs toward the force direction on PDMS. Adapted with permission from ref (75). Copyright 2011 Springer Nature. (g) Piezoelectric ZnO nanoparticles enable mechano-cross-linking of thiol–ene components, which leads to improved mechanical properties. (h) Mechanochemically trained organo-gel showed better shape retention and larger stress-to-strain compared to the untrained gel. (i) Force, vibrational frequency, and time as different input factors that lead to adaptation of storage modulus of the trainable organogel. Panels g–i are adapted with permission from ref (78). Copyright 2021 Springer Nature.

Inspired by structural organization that enables mechanical adaptation in biological tissues, numerous biomimetic systems and mechano-guided designs take advantage of the reorganization capabilities of nanostructures to enable mechanical training.^16−19,70^ With repeated prestretching, Lin *et al*. found that the randomly oriented nanofibers and nanocrystalline domains of a poly(vinyl alcohol) hydrogel gradually aligned toward the direction of applied stress. The mechanical training produced muscle-like properties including a low Young’s modulus, a high fatigue threshold, and high strength.^71^ Song *et al*. synthesized a polyamide elastomer with an amorphous matrix of nanocrystalline domains.^72^ Following step-cycle tensile deformation, the crystalline domains were oriented and aligned along the tensile direction, resulting in an over 7-fold increase in tensile strength, compared to the untrained elastomer (Figure 4a,b). In another study on artificial muscle, Zn^2+^-based sacrificial coordination bonds in a polyolefin elastomer facilitated alignment of chain segments along the force direction with cyclic rupture and reconstruction, leading to strain-induced crystallization and stiffening.^73^ Our group has developed a tissue-like composite that mimics cell/ECM interactions using starch granules/polyacrylamide–alginate.^74^ By stretching the composite material at different angles, we can dynamically reconfigure both the packing and orientation of the granular network through its interaction with the nanostructured hydrogel matrix (Figure 4c), resulting in a mechanical memory device whose strain-history is dependent on stress–strain hysteresis (Figure 4d,e).

Materials with trainable electrical properties can be designed by leveraging the strain-induced alignment of nanostructures. Lipomi *et al*. fabricated skin-like pressure and strain sensors by spray coating carbon nanotubes (CNTs) on polydimethylsiloxane (Figure 4f). During stretching, the CNT bundles aligned with the axis of strain, while during relaxation, the nanotubes buckled in the direction of stretching. With repetitive cyclic loading, the resistance of the CNT thin films decreased based upon strain history.^75^

Additionally, like biological systems, mechanically adaptable biomimetic materials may assemble simple building blocks, often through mechanochemical reactions, into complex structures when stimulated.^76^ To mimic the regrowth of damaged skeletal muscle after training, Matsuda *et al*. constructed double-network hydrogels containing building-block monomers.^77^ Mechanical disruption of the brittle polymer chains in the double-network hydrogels generated mechanoradicals, which triggered polymerization of the monomers in the matrix. Through repeated mechanical training, new networks formed to replace brittle ones, resulting in augmented mechanical properties. The materials system also demonstrated self-growth in size and strength over time when enough building blocks were present, similar to the uptake of amino acids and other building materials by muscle tissues for regrowth. Nanoporous networks with high permeability can ensure the kinetics required for exchange of materials. Thus far, we have discussed mechanically trainable systems based on direct mechanical manipulation, such as stretching. Would it be possible to develop materials that can receive mechanical training wirelessly? Using piezoelectrically induced cross-linking, Wang *et al*. developed a trainable material by embedding ZnO nanoparticles and using ultrasound to create mechanical stress, which results in cross-linking of thiol–ene components (Figure 4g).^78^ Mechanical training showed evidence of strengthening (Figure 4h), which could also be mediated by force magnitude, frequency, and time (Figure 4i). Multimodal trainable systems capable of responding to multiple dimensions of stimuli allow materials to cope with various complex environments upon training.

#### Electronic Trainable Systems, Memristors, and Neuromorphic Computing

**Figure 5:**
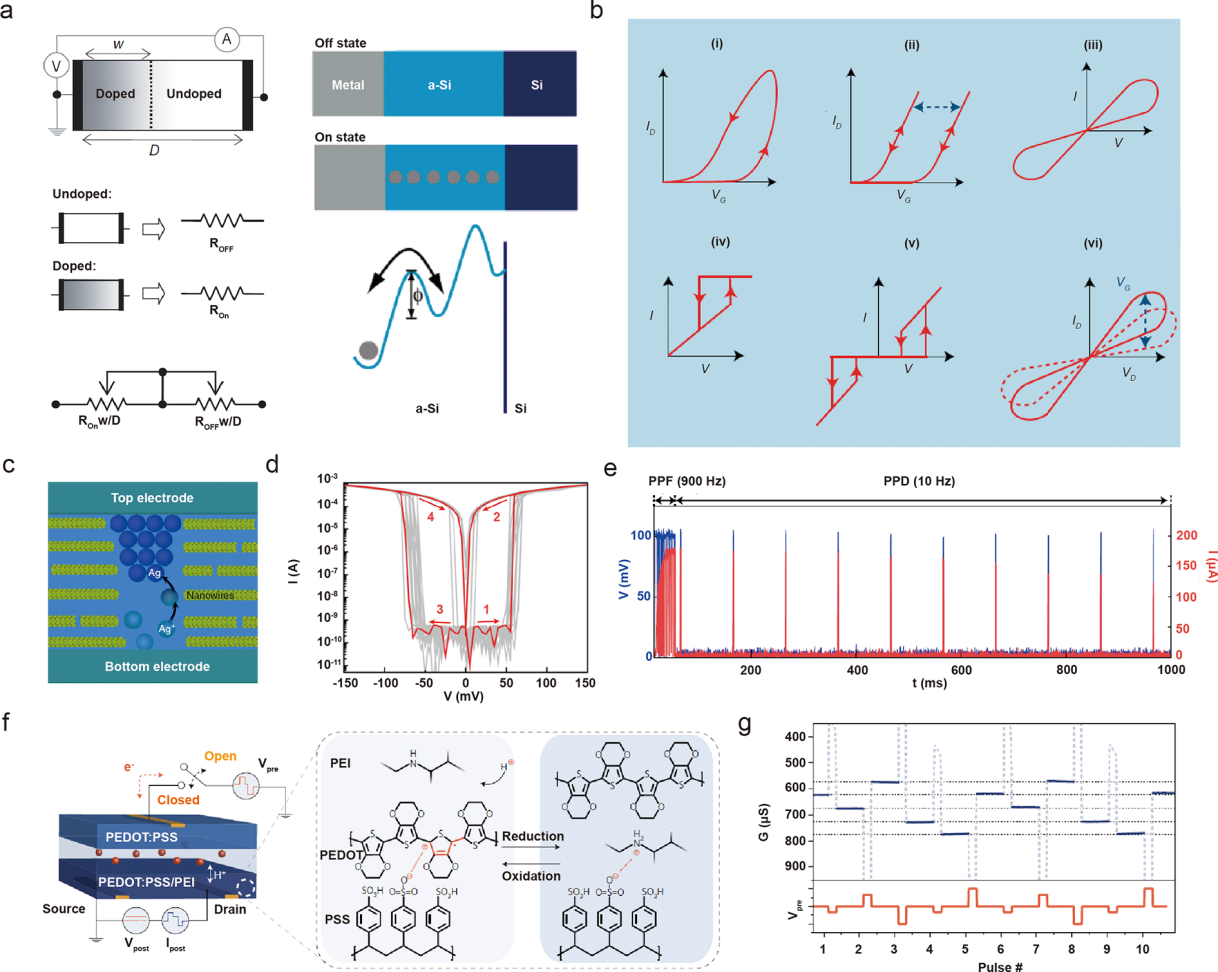
Electrically reconfigurable nanostructured memristors. (a) Two fundamental memristor designs. (left) Valence change memories. (right) Formation of conductive filaments. Adapted with permission from ref (79). Copyright 2008 Springer Nature. (b) Memory shown by the hysteresis in *I*–*V* curves in current memristor devices. Adapted with permission from ref (80). Copyright 2020 Springer Nature. (c) Illustration of conductive pathway formation in a protein nanowire catalyst upon applied voltage. (d) *I*–*V* curves showing the resistance switch at <100 mV applied voltage. (e) Artificial synaptic conditioning with 100 mV and 1 ms electric pulse input. Panels c–e are adapted with permission under a Creative Commons CC BY License from ref (86). Published 2020 by Springer Nature. (f) Illustration of the organic electrochemical memristor and conductance switching mechanisms via redox reactions at PEDOT:PSS/PEI electrode in the postsynaptic electrode. (g) Nonvolatile switching behavior shown by discrete conductance states. Panels f and g are adapted with permission from ref (89). Copyright 2017 Springer Nature.

In 2008, Stanley Williams invented the “memory resistor” or “memristor”, realizing the prototype for electronic trainable systems.^79^ The preliminary device consists of a layer of 5 nm doped TiO_2–*x*_ and undoped TiO_2_, sandwiched between parallel electrodes. Using a voltage bias, oxygen vacancies migrate from doped TiO_2–*x*_ to undoped TiO_2_, producing a nonvolatile resistive switch essential for forming logic gates and characterized by on/off ratios (Figure 5a). The memristor is widely accepted as reverse engineering of synaptic potentiation in the brain. It allows for training and enhancement of specific material properties (e.g., resistance) through external stimulation (e.g., voltage) as well as memory, as demonstrated by hysteresis loops in the *I*–*V* curve^80,81^ (Figure 5b). Since the trainable device is capable of altering weights by modifying resistance by applied voltage, it could revolutionize current digital computers, which make decisions based on 0s and 1s and would ultimately lead to neuromorphic computing hardware.^82^ The prototypical memristor has substantially impacted nanomaterial and nanoionic-based research on artificial synapses.^81^ Some of the initial attempts at artificial synapses involved making solid-state devices consisting of nanoscale conductive and nonconductive domains, categorized into two types. In the first type, electrochemically active metals (e.g., Ag) are used to produce conductive filaments or dendrites in semiconducting or insulating media (e.g., Si, SiO_2_, and SiGe), which can then be used for memory storage (Figure 5a, right). In the second type, oxygen-vacancy diffusion is exploited to create valence change memories in nanometer-thick oxide layers (Figure 5a, left).^83^ Additionally, combinations of the two have been demonstrated in transition metal oxide layers such as TaO_*x*_, HfO_*x*_, and TiO_*x*_.^84^ The collection of resistance switching mechanisms has grown over time with the addition of low dimensional materials, redox-active polymers, ferroelectric materials, and spintronics.^85^ There have also been attempts to match the properties of biological systems in terms of low voltage configurations and fast temporal integration (e.g., ∼100 mV, ∼10 ms). Developing a nanoscale catalyst that is capable of lowering the metal reduction overpotential appears to be a promising approach. Fu *et al*. used protein nanowires from the bacterium *Geobacter sulfurreducens* as the solid electrolyte layer to enhance Ag^+^ reduction kinetics (Figure 5c). A voltage bias <100 mV has been demonstrated for switching, which mimics the biovoltage in synaptic transmissions (Figure 5d). Furthermore, artificial conditioning has been achieved using 100 mV input voltage and 1 ms frequency, indicating great promise for integration into biological systems (Figure 5e).^86^

**Figure 6:**
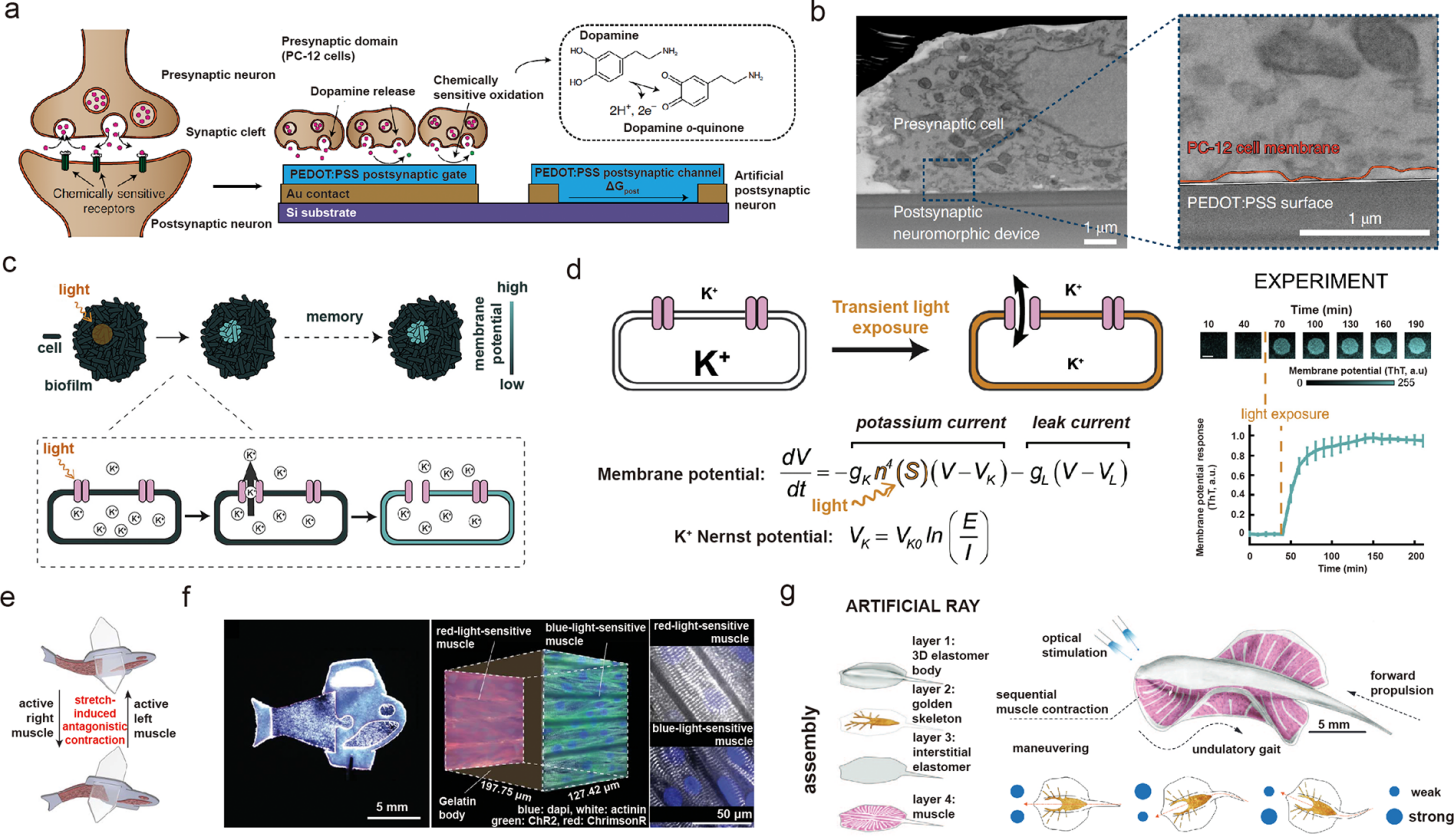
Biohybrid composites and engineered living materials (ELMs) for future trainable systems. (a) Schematic illustration of a biohybrid synapse. (b) SEM images showing the synaptic junction between neurons and the postsynaptic electrode. Panels a and b are adapted with permission from ref (90). Copyright 2020 Springer Nature. (c) Incident irradiation on *B. subtilis* biofilm with light results in hyperpolarization of bacterial membrane potential that persists even after light is removed. (d) Memory encoded in the membrane potential persists for hours, resulting in the antiphase correlation in the naturally fluctuating membrane potential between the illuminated (bright region at 0 h) and unilluminated region of the biofilm. Panels c and d are adapted with permission from ref (101). Copyright 2020 Elsevier. (e) Illustration of biohybrid fish movement by antagonistic design. (f) Optical images of biohybrid fish and biological components from panel e. Panels e and f are adapted with permission from ref (103). Copyright 2022 American Association for the Advancement of Science. (g) Illustration of the bioinspired ray that can be guided by optical stimulation. Adapted with permission from ref (102). Copyright 2016 American Association for the Advancement of Science.

Organic electrochemical transistors (OECTs) differ from inorganic transistors in that they utilize ionic charge injections from the electrolyte to modulate the doping state of the organic semiconductor. Considering that ionic penetration is a volumetric effect, organic transistors may achieve greater capacitance than inorganic transistors with parallel-plate configurations. Despite their high transconductance, OECTs suffer from a kinetic limitation that is dominated by ionic circuits. However, the low power consumption and ionic nature of OECTs make them promising for mimicking natural synaptic processes and are highly biocompatible for use in biohybrid systems.^87^ Based on printable OECTs, Harikesh *et al*. reported organic electrochemical neurons that produce action potentials. Additionally, the neuromorphic device demonstrated biointegration with the Venus flytrap, modulating its response by current injection.^88^ van de Burgt *et al*. demonstrated a low-voltage and nonvolatile artificial synaptic interface using PEDOT:PSS as the presynaptic electrode and PEI/PEDOT:PSS as the postsynaptic electrode.^89^ After receiving the applied potential, PEI in the postsynaptic electrode got protonated, and electrons flew through external circuits to scavenge holes in the PEDOT backbone, causing resistance switching (Figure 5f). Additionally, the artificial synaptic interface demonstrated nonvolatile conductance switching with discrete conductance states with overwrite electric pulses, showing great promise for reliable neuromorphic devices (Figure 5g). This artificial synaptic device overcame the kinetic limitations of previously reported organic memristors that used ionic diffusion. As a follow-up study, a biohybrid synapse was demonstrated by directly coupling dopaminergic cells to an OEC device, where dopamine oxidation at the gate electrode led to an increase in postsynaptic channel conductance and synaptic conditioning (Figure 6a).^90^ The success of the biohybrid synapse is largely due to the coupling between dopaminergic cells and PEDOT:PSS electrodes; the gap of ∼100 nm between the two components mimics that of the biological synaptic cleft (Figure 6b). Recently, a stretchable neuromorphic nerve was integrated with a paralyzed mouse to restore its motor function. Artificial synapses provided proprioceptive senses and enabled feedback loops for neurorehabilitation applications.^91^ Taking a step further, artificial synaptic interfaces have been utilized as essential components for biological and robotic control, through mechanically, optically, and chemically mediated communication loops. The future of artificial synapse frontiers will be advanced by systematic engineering that bridges material science, bioengineering, and artificial intelligence.^92−94^

## Future Directions and Outlook

Nature’s adaptable processes can inspire a vast range of material designs and biointerface applications. In the sections above, we reviewed nanostructured materials and devices that enabled biochemical, mechanical, and electrical trainable systems. Despite many promising proof-of-concept results, there are limitations and challenges which require future improvements. For example, nanomaterials can sometimes suffer from compromised stability. Local stresses, elevated temperatures, and reactive chemical species within biological environments may exacerbate the instability. Thus, materials selection and surface modification for trainable nanomaterials should be rationally considered. In addition, nanoenabled approaches present a challenge for *in vivo* training due to low targeting efficiency and the complexity of the *in vivo* environment. However, mitigation strategies from the nanomedicine field may be helpful. Moreover, little attention has been paid to the extraction of nanomaterials after training, which is a technical challenge. Some of these issues could be addressed by the development of on-demand biodegradable or bioresorbable nanomaterial platforms. Finally, the training is not normally reversible since it induces permanent changes to the materials, such as polymerization or cross-linking. Enhancing the programmability and dynamic adaptability of biomimetic systems could lead to greater opportunities.

In the quest for seamless biointerfaces, the intracellular self-assembly of functional nanomaterials presents a promising option.^95^ By exploiting the different physicochemical environments found in different cellular compartments, for instance, pH, ionic concentration, or enzymes, we can design functional nanomaterials that self-assemble *in situ* for direct subcellular training with high selectivity. In addition, training protocols must be carefully designed to uncover the fundamental mechanisms and kinetics of biological adaptation. Cardiomyocytes, for instance, can adapt to both subthreshold and suprathreshold electrical stimulation to establish synchronized pacing. The same behavior may be observed in other cell types that respond to a variety of stimuli. Researchers should examine the time scales on which adaptivity develops within cells, as well as the phenotypic heterogeneities within and between cells. It is also important to consider whether training is conducted *in vitro* or *in vivo*, as environment, such as surrounding ECMs and other coupled cells, can change the training threshold and the establishment of trained behavior. Characterization of subcellular structures using advanced imaging techniques may address fundamental questions. Furthermore, because biological training is a long-term process, the incorporation of artificial intelligence into trainable systems will facilitate analysis of the large amounts of generated data. Computer vision techniques can be used to count and analyze cells automatically.^96^ Our lab uses a computer vision-based detection system to collect information from cardiomyocytes following long-term electrical training as part of our ongoing research project. As such, high-throughput screening could be used to decipher “bioelectromics” data. Decision-making through machine learning enables closed-loop response systems, which are able to program training protocols that learn from cell fates. Ultimately, this approach will allow us to identify the principles and key parameters underlying cellular adaptability. Ascending the biological hierarchy, cell adaptation leads to tangible and macroscopic changes such as stiffening of bones or addictive behavior in patients. Thus, to translate our understanding of cellular adaptability into meaningful real-life applications, we must bridge how cell training could alter behavior at the tissue, organ, and organism levels.

Our current understanding of how materials modulate and train living systems through various energy transduction pathways leads us to ask if cells in reverse could “train” the materials as a whole? The answer lies in the emerging field of synthetic biology-guided biomaterial synthesis and ELMs. With the advent of synthetic biology, it is now possible to precisely engineer cells or even engineer cell-free expression (CFE) systems, which respond to outside stimuli to yield hierarchical biomaterials with programmable structure and functionality.^4,97^ In one study, Liu *et al*. reported the polymerization of conductive polymers such as PANI, PEDOT, and PDAB from precursor reagents within the cellular membrane of genetically modified neuron cells with humanized ascorbate peroxidase Apex2 genes, indicating a route to precise positioning and integration of nanomaterial–bioelectrical interfaces.^97^ DNA technology is fueling progress in functional nanomaterial assembly, and almost limitless geometric arrangements of nanoparticles are now possible with programmed DNA hybridization.^98^ In the ELM platform, microbial cells are used to reprogram the properties of materials through reinforcement, degradation, or the functionalization of their matrix, yielding an adaptable material platform that responds to environmental stimuli.^99^ We envision a living material that retains the memory of previous stimulation and responds more aggressively to subsequent stimulation. A potential benefit of this approach, that integrates the principles of biointerface training with the ELM platform, is that living materials may be trained prior to their deployment (similar to adoptive T-cell therapy) to enhance their functionality. An encapsulated whole-cell biosensor that retains memories of previously encountered analytes will yield improved sensitivity or specificity upon re-encounter. We can also imagine the formation of spatiotemporally controlled patterns in an ELM using cells that remember and re-exhibit patterns when re-stimulated. Targeting adaptive and memory-forming molecular components or introducing synthetic genetic circuitry would enable the writing and erasing of cell-encoded memory in ELMs.^100^ It is possible, for example, to encode memory in the membrane potential of bacteria for many hours (Figure 6c,d).^101^

The use of functional nanomaterials in ELMs, a feature we believe essential for trainable ELMs, has been scarcely explored in previous studies. An earlier section discussed the use of stimuli-responsive nanomaterials and their direct interaction with mammalian cells to enable effective cell training. Since most microorganisms have a size well matched for nanomaterials, we posit that nanomaterials can provide highly efficient training. It is also feasible to engineer biohybrid robotic systems, where materials are moved by the force exerted by cells. Both autonomous and stimulus responsive microrobots have been demonstrated (Figure 6e–g),^102,103^ and we anticipate that training will enable these systems to accomplish programmable and adaptable locomotion, such as mimicking swarm-like synchronous motion for camouflage or hyperresponsiveness to escape from danger. We believe that nanomaterial-enabled training of living materials will yield functions and modalities that are not achievable from their individual parts.
